# Investigation of Eumelanin Biosynthesis in *Gluconacetobacter tumulisoli* FBFS 97: A Novel Insight into a Bacterial Melanin Producer

**DOI:** 10.3390/microorganisms13030480

**Published:** 2025-02-21

**Authors:** Jiayun Song, Yanqin Ma, Zhenzhen Xie, Fusheng Chen

**Affiliations:** 1 College of Food Science and Technology, Huazhong Agricultural University, Wuhan 430070, China; jiayunsong@webmail.hzau.edu.cn (J.S.); mayq@webmail.hzau.edu.cn (Y.M.); 13683977281@163.com (Z.X.); 2 National Key Laboratory of Agricultural Microbiology, Huazhong Agricultural University, Wuhan 430070, China; 3 Hubei International Scientific and Technological Cooperation Base of Traditional Fermented Foods, Huazhong Agricultural University, Wuhan 430070, China; 4 School of Life Sciences, Guizhou Normal University, Guiyang 550025, China

**Keywords:** *Gluconacetobacter tumulisoli* FBFS 97, melanin, eumelanin, biosynthetic pathway, acetic acid bacteria

## Abstract

Acetic acid bacteria (AAB) are a group of bacteria, most of which can produce pigments. However, the mechanism of pigment production by AAB is unclear. A strain of AAB, *Gluconacetobacter tumulisoli* FBFS 97, which can produce a large amount of brown pigment (BP), was isolated in our previous research. In the current study, it was found that the BP yield of the FBFS 97 strain was enhanced in the presence of tyrosine, and an intermediate of melanin, L-3,4-dihydroxyphenylalanine (L-DOPA), was identified using ultra-performance liquid chromatography–quadrupole time-of-flight mass spectrometry (UPLC-Q-TOF-MS). The structural properties of BP were analyzed by pyrolysis gas chromatography–mass spectrometry (Py-GC-MS). All these analyses suggest that BP may be eumelanin, a type of melanin. Then, the eumelanin biosynthetic pathway was investigated in the FBFS 97 strain, and three related genes with eumelanin including *pheA*, *yfiH*, and *phhB* in its genome were found and knocked out, respectively. The results showed that eumelanin production increased 1.3-fold in the *pheA* deletion mutant compared to the wild-type FBFS 97 strain, but when either *yfiH* or *phhB* was knocked out, the eumelanin production in the mutants was the same as that in the wild-type FBFS 97 strain. Finally, a possible biosynthetic pathway for eumelanin in the FBFS 97 strain is proposed.

## 1. Introduction

Melanin, a group of natural dark pigments, is a heterogeneous polymer derived from the oxidation and polymerization of phenolic or indole compounds, widely present in animals, plants, and microorganisms [1,2,3]. Melanin is commonly used in cosmetics [4], functional foods [5], semiconductor materials [6], radiation-resistant clothing [7], and eye masks due to its ultraviolet absorption capacity [8], dye properties [9], semiconductor characteristics [6], and radiation resistance [10]. Compared with melanin from plants and animals, microbial melanin, especially from bacteria such as the genera of *Streptomyces*, *Pseudomonas*, *Azotobacter*, and *Rhizobium* [11], is easy to produce with high cost-effectiveness [12], so bacterial melanin has attracted more and more attention.

Up to now, some biosynthetic pathways of bacterial melanin have been investigated, mainly including the L-3,4-dihydroxyphenylalanine (L-DOPA) pathway in which eumelanin and pheomelanin are formed from L-DOPA, and the homogentisic acid (HGA) and 4-hydroxyphenylacetic acid (4-HPA) pathways in which pyomelanin and 4-hydroxypheny-lacetic acid melanin are produced from the HGA and the isomer 3, 4-dihydroxyphenylacetic acid, respectively [13,14,15,16] (Figure S1). Among them, in the L-DOPA pathway of melanin biosynthesis, tyrosinase (EC 1.14.18.1) is a key enzyme [13,17]. However, when tyrosinase is absent in microbial cells, laccase (EC 1.10.3.2) becomes the key enzyme for L-DOPA melanin biosynthesis [18]. Recently, a laccase-like protein YfiH and a catalase CatA have been proven to be the key enzymes for L-DOPA melanin biosynthesis in *Aeromonas media* WS [19]. Moreover, other proteins can also regulate L-DOPA melanin production in *Aeromonas* spp. For instance, a PilF protein, an outer membrane protein essential for flagellar biosynthesis in *A. veronii* B565, has been implicated in regulating melanin production [20]. These findings suggest that the bacterial melanin biosynthetic pathways are various and more complex than previously thought.

Acetic acid bacteria (AAB), a group of bacteria, can produce pigments that are commonly used as taxonomic indicators [21]. However, there is very little research on the pigment biosynthesis pathway of AAB. Recently, we isolated an AAB strain, *Gluconacetobacter tumulisoli* FBFS 97, which can produce a large amount of brown pigment (BP), most likely melanin [22]. In this research, the effects of carbon sources, tyrosine, phenylalanine, and vitamin C on BP production by the FBFS 97 strain were investigated. A melanin intermediate, L-DOPA, was identified, and the BP was further characterized as eumelanin, a type of melanin, through comprehensive analysis. Then, the eumelanin biosynthesis pathway in the FBFS 97 strain was explored. The results showed that the FBFS 97 genome lacks the tyrosinase gene but contains a laccase-like gene, *yfiH*. Knockout of the *yfiH* gene did not affect eumelanin production. Next, considering that tyrosine is a precursor for eumelanin synthesis, two key genes in the tyrosine biosynthesis pathway, *phhB* (which converts phenylalanine to tyrosine) and *pheA* (which catalyzes the conversion of prephenate to phenylpyruvate), were individually knocked out. The results showed that the knockout of *phhB* did not affect eumelanin production. However, the knockout of *pheA* resulted in a 1.3-fold increase in eumelanin production compared to the wild-type strain. Based on these results, an alternative tyrosinase-independent pathway for eumelanin biosynthesis is proposed. Our study provides a novel clue for further investigation into the melanin biosynthetic pathway in AAB strains.

## 2. Materials and Methods

### 2.1. Bacterial Strains and Culture Conditions

*G. tumulisoli* FBFS 97 was preserved in our laboratory [22]. *Escherichia coli* DH5α was purchased from Beijing Quanshi Gold Biological Co., Ltd. (Beijing, China). The plasmid pKOS6b was presented by Prof. Wolfgang Liebl from the Technical University of Munich, Germany [23].

The FBFS 97 strain was cultivated in the glucose and yeast extract (GYE) seed medium containing 0.1% D-glucose and 1% yeast extract, or in the GYE fermentation medium containing 4% D-glucose and 1% yeast extract. If a solid medium is required, 1.5% agar was added. The pH values of the media were adjusted to 6 using 0.5 M hydrochloric acid (HCl). Transformed *Escherichia coli* DH5α, harboring the plasmid pKOS6b, was cultivated in Luria–Bertani (LB) medium (1% tryptone, 0.5% yeast extract, 1% NaCl) at 37 °C for 12 h, and if necessary, 50 μg/mL kanamycin was added.

### 2.2. Effects of Carbon Sources and Time-Course Fermentation on BP Production of the FBFS 97 Strain

To improve BP production, the media, respectively, including 2% (*w*/*w*) D-mannitol, D-glucose, sucrose, D-lactose, D-fructose, D-maltose or glycerol, and 1% yeast extract, were prepared. Then, 1% (*v*/*v*) FBFS 97 seed broth, which was incubated for 24 h at 30 °C and 150 rpm on the shaker (HZ200LBG, Wuhan Ruihua Instrument & Equipment Co., Ltd., Wuhan, China), was inoculated in the above-mentioned media, respectively, and cultivated at 30 °C and 150 rpm. After six days of cultivation, the broth of the FBFS 97 strain was centrifuged for 10 min at 8000 rpm (Neofuge 18R, Heal Force Development Ltd., Hong Kong, China). The BP content in the supernatant was measured by an ultraviolet–visible (UV-Vis) spectrophotometer (UV-2600, Shimadzu Corporation, Kyoto, Japan) at 440 nm [24], and the precipitate was dried using a freeze dryer (FDB-5 Freeze Dryer, Gold-SIM Corporation, Seattle, WA, USA) and weighed to assess the growth of the FBFS 97 strain. Finally, the effects of glucose concentrations at 2%, 4%, 6%, 8%, 10%, 12%, and 14% on BP production were analyzed, respectively.

To maximize BP production, liquid fermentation was conducted under the optimal glucose concentration, and the BP yield and biomass were measured every 2 days during fermentation.

### 2.3. Effects of Tyrosine, Phenylalanine, and Vitamin C on BP Production of the FBFS 97 Strain

The FBFS 97 strain was inoculated and incubated in the GYE seed medium for 18 to 24 h, after which the broth was transferred to separate GYE fermentation media, each containing phenylalanine (0.5–2.5 mM), tyrosine (0.5–2.5 mM), or vitamin C (0–25 mM), with an inoculation rate of 1% (*v*/*v*), respectively. The culture was then incubated at 30 °C and 150 rpm until the sixth day. Afterward, the broth was centrifuged at 8000 rpm to remove the bacterial cells, and the BP content in the supernatant was detected using the UV-Vis spectrophotometer (UV-2600, Shimadzu Corporation, Kyoto, Japan).

### 2.4. L-DOPA Produced by the FBFS 97 Strain Was Detected Through UPLC-Q-TOF-MS

An amount of 1 mL of freshly prepared FBFS 97 strain culture in GYE seed medium was incubated at 30 °C for 24 h, then added to 50 mL of GYE fermentation medium in a 250 mL Erlenmeyer flask and incubated at 30 °C with shaking at 150 rpm for 6 days. The supernatant was then collected by centrifugation at 8000 rpm for 15 min and filtered through a 0.22 µm membrane (Tianjin Jinteng Experimental Equipment Co., Ltd., Tianjin, China) for UPLC-Q-TOF-MS analysis with a Waters Vion IMS QTof (Waters Corporation, Milford, MA, USA).

Then, 1 µL of the filtered supernatant was injected into UPLC-Q-TOF-MS with a Waters ACQUITY UPLC-BEH C18 column (1.7 µm, 2.1 mm × 100 mm) at 32 °C, and the flow rate was established at 0.3 mL/min with 90% (*v*/*v*) 10 mM acetic acid and 10% (*v*/*v*) methanol as a mobile phase for L-DOPA detection in negative electrospray ionization mode (ESI^−^), compared with the L-DOPA standard (CAS: 59-92-7, Aladdin, Shanghai, China).

### 2.5. Extract and Characterization of BP Produced by the FBFS 97 Strain

#### 2.5.1. Extract and Characterization of BP Produced by the FBFS 97 Strain

**Figure 1:**
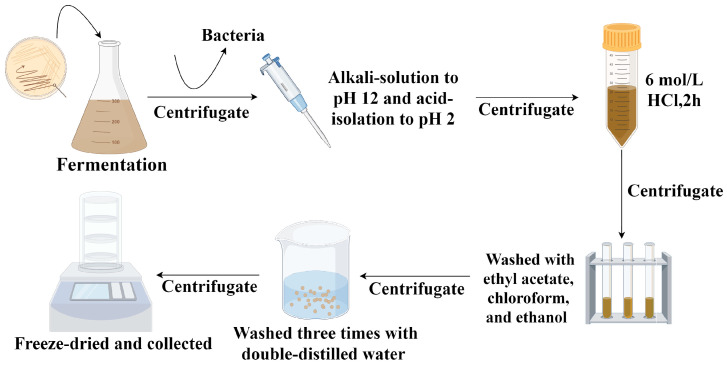
Extraction and purification of BP produced by the FBFS 97 strain. This figure was created using Figdraw 2.0.

The extraction and purification of BP were conducted following the method described by Guo et al. (2014) [25], with some slight modifications. The specific steps (Figure 1) are as follows: the broth, after 10 days of fermentation, was centrifuged at 10,000 rpm for 10 min to obtain the supernatant, which was then filtered through sterile 0.22 µm membranes for sterilization. All subsequent steps were carried out under sterile conditions to prevent contamination or spoilage. The pH of the supernatant was adjusted to 2 using 0.5 M HCl and left at 25 °C in the dark for one week. After this period, the mixture was centrifuged at 10,000 rpm for 10 min to obtain the crude BP precipitate, which was then dissolved in 0.5 M NaOH, and the pH was adjusted to 12 using 0.5 M NaOH, followed by further adjustment to pH 2 with 0.5 M HCl. Subsequently, the pre-purified BP precipitate was collected by centrifugation at 10,000 rpm for 10 min. To further purify BP, the pre-purified BP precipitate was suspended in 6 M HCl for 2 h, followed by centrifugation and washing with distilled water until the pH value became neutral. The precipitate was sequentially washed with ethyl acetate, chloroform, and ethanol to remove lipids and other residues. Finally, the precipitate was washed three times with double-distilled water, centrifuged, collected, and freeze-dried to a constant weight to obtain the purified BP.

The purified and freeze-dried BP powder was dissolved in 0.1 M NaOH. The UV-Vis spectrum of the BP solution was recorded in the wavelength range of 200–800 nm using the UV-Vis spectrophotometer, with 0.1 M NaOH used as the blank control.

#### 2.5.2. Thermal Characterization of BP

Thermogravimetric analysis (TGA) was conducted using a TGA 4000 instrument (PerkinElmer, Norwalk, CT, USA) to assess the thermal stability of BP, following the methodology outlined by [26]. Approximately 4 mg of the purified BP powder was heated from 30 °C to 800 °C in nitrogen at a heating rate of 20 °C/min, and the thermogravimetric (TG) and derivative thermogravimetric (DTG) curves of BP were recorded.

#### 2.5.3. Structural Characterization of BP

A pyrolysis gas chromatography–mass spectrometry (Py-GC-MS) system was used to characterize the BP structure, following the approach described in [27]. The system mainly includes a thermal cracker EGA/PY-3030D (Frontier Corporation, Tokyo, Japan), an analytical instrument GC/MS-QP2010 Ultra (Shimadzu Corporation, Japan), and a UA-5MS column (30 m × 0.25 mm × 0.25 μm). The analysis was performed under a pyrolysis temperature of 650 °C for 12 s, an interface temperature of 300 °C, and a GC injection port temperature of 320 °C, with a split ratio of 30:1 and a column flow rate of 1 mL/min. The GC oven temperature was programmed from 40 °C (isothermal for 1 min) to 80 °C at a rate of 5 °C/min, then increased at a rate of 15 °C/min to 300 °C (isothermal for 15 min). The electron energy was set to 70 eV, with an ion source temperature of 230 °C, a transfer line temperature of 300 °C, and a mass scan range of *m*/*z* 40–550 in full scan mode.

### 2.6. Prediction of the Related Genes with Melanin Production in the FBFS 97 Strain

To investigate if the FBFS 97 strain possesses a pathway for metabolizing tyrosine into melanin, the core conserved domains of tyrosinase (pfam00264) and laccase (pfam00394, pfam07731, pfam07732, and pfam02578) were scanned in the FBFS 97 strain’s genome using HMMER software 3.2.2. The homologous genes of Phenylalanine hydroxylase (PhhA) and Pterin-4-α-methanolamine dehydratase (PhhB), which are involved in the conversion of phenylalanine to tyrosine, and Prephenate dehydratase (PheA), which catalyzes the conversion of prephenate to phenylpyruvate, were analyzed in the FBFS 97 strain’s genome using the NCBI’s BLASTP (https://blast.ncbi.nlm.nih.gov/Blast.cgi, accessed on 15 November 2023) and the KEGG pathway databases (https://www.kegg.jp/, accessed on 15 November 2023).

### 2.7. Effects of the Melanin-Relative Genes on Melanin Production in the FBFS 97 Strain

Based on the prediction result in Section 2.6, the melanin-related genes in the FBFS 97 strain were deleted using the efficient markerless gene deletion system described by Peters et al. (2013) [28]. Specifically, in principle, the suicide plasmid pKOS6b was constructed using Cytosine deaminase (CodA) and Cytosine permease (CodB) from *Escherichia coli* reverse screening markers. PKOS6b integrated the entire plasmid into the target genome in the first homologous recombination, followed by a second allelic exchange using the reverse screening markers under the stress of the exogenous environment, resulting in the entire plasmid being detached from the genome and removed from the cell due to the inability to replicate, ultimately producing a trace-free knockout deletion strain. The primers, which were used to construct the gene deletion vectors, are listed in Table S1.

The melanin contents in the supernatants of the FBFS 97 strain and its gene-deleted strains were detected at 440 nm every day until the 6th day by the UV-Vis spectrophotometer. Their biomasses were measured at 600 nm every 6 h for 48 h.

### 2.8. Statistical Analysis

Analysis data were expressed as means ± standard errors from three replicates. Significance was assessed by one-way analysis of variance (ANOVA) using the SPSS 26 (International Business Machines Corporation, Armonk, NY, USA).

## 3. Results

### 3.1. Regulation of BP Production and L-DOPA Detection in the FBFS 97 Strain

#### 3.1.1. Effects of Carbon Sources and Time-Course Fermentation on Growth and BP Production of the FBFS 97 Strain

**Figure 2:**
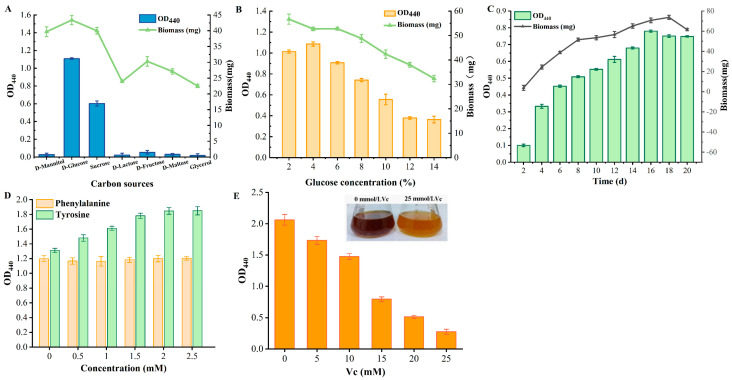
BP yield and biomass of FBFS 97 strain under different carbon sources (**A**), different glucose concentrations (**B**), and different fermentation days (**C**), as well as the effects of different concentrations of tyrosine and phenylalanine (**D**) and vitamin C (Vc) (**E**) on BP production by FBFS 97 strain.

*G. tumulisoli* FBFS 97, which can produce a large amount of BP, was isolated in our previous research [22]. To optimize its growth and BP production, the effects of carbon sources including D-mannitol, D-glucose, sucrose, D-lactose, D-fructose, D-maltose, and glycerol on the growth and BP yield of the FBFS 97 strain were detected, and the results were showed that D-glucose was the best carbon source, followed by sucrose. Glycerol, however, was the least effective carbon source for the growth and BP production of the FBFS 97 strain (Figure 2A).

Then, the effects of D-glucose at different concentrations (2%, 4%, 6%, 8%, 10%, 12%, and 14%) on BP yields were investigated, and the results (Figure 2B) revealed that 4% D-glucose resulted in the highest BP production. Therefore, the medium containing 4% D-glucose and 1% yeast extract was used in the following experiments.

As shown in Figure 2C, fermentation changes over time were observed. The biomass of the FBFS 97 strain steadily increased, reaching its maximum on day 18. A noticeable color change in the broth was observed starting from day 4, gradually darkening, indicating that BP accumulated from that point onward. The BP content continued to increase from day 4 (OD_440_ = 0.332 ± 0.0121) to day 16 (OD_440_ = 0.780 ± 0.0065), peaking on day 16.

#### 3.1.2. Effects of Tyrosine, Phenylalanine, and Vitamin C on BP Production of the FBFS 97 Strain

To determine whether BP is related to melanin, we first investigated factors related to melanin production, such as tyrosine, phenylalanine, and vitamin (Vc), to explore if they affect BP production. Tyrosine is commonly used as a precursor in melanin biosynthesis through the melanin biosynthetic pathway [29], while phenylalanine is converted to tyrosine to enhance melanin production. Additionally, Vc, acting as an antioxidant, can reduce the tyrosinase product DOPA quinone to L-DOPA, thereby inhibiting the biosynthesis of L-DOPA melanin [30].

As illustrated in Figure 2D, when tyrosine was added to the media, the OD_400_ values were higher at all concentrations compared to those when phenylalanine was added, indicating that tyrosine had a stronger promoting effect on BP production than phenylalanine. In addition, the OD_440_ values showed an upward trend, especially between 0 mM and 1.5 mM, where the increase in tyrosine concentration led to a significant rise in OD_440_ values. Then, the OD_440_ values tended to stabilize at concentrations of 2 mM and 2.5 mM, suggesting that BP production reached saturation. In contrast, the addition of phenylalanine to the medium had no significant effect on BP production by the FBFS 97 strain, indicating that tyrosine is the more effective substrate for BP production. What is more, it was observed that as the concentration of Vc increased, the OD_440_ value showed a clear decreasing trend. Specifically, when Vc was not added to the GYE fermentation media, the OD_440_ value was the highest, and it decreased significantly as the Vc concentration increased, reaching the lowest point at 25 mM (Figure 2E). This observation is consistent with previous studies on the effects of Vc on melanin production in *Shewanella algae* [31].

#### 3.1.3. L-DOPA Detected by UPLC-Q-TOF-MS

To evaluate the potential of the FBFS 97 strain for melanin production, L-DOPA, a key precursor in melanin biosynthesis [32], was analyzed by UPLC-Q-TOF-MS in the fermentation broth of the FBFS 97 strain.

**Figure 3:**
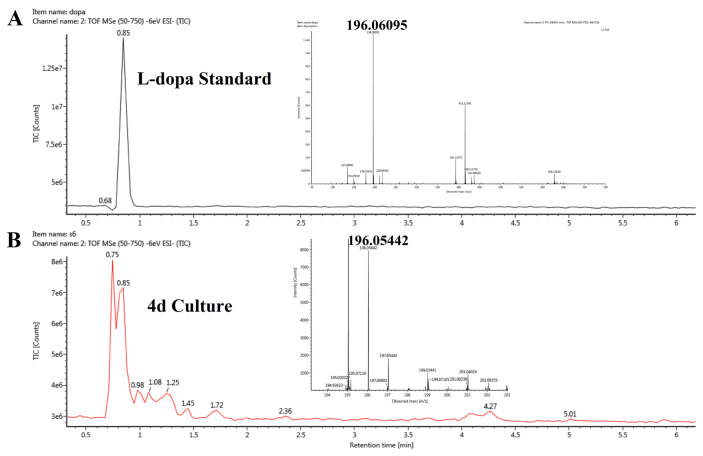
Detection of L-DOPA in FBFS 97 culture by UPLC-Q-TOF-MS. L-DOPA standard (**A**) and L-DOPA in fermentation broth on the fourth day (**B**).

As shown in Figure 3A, the peak for the L-DOPA standard appeared at 0.85 min with a molecular weight of 196.06095 in negative ion mode. No corresponding peak was detected in the fermentation broth on days 1 to 3, but a peak at 0.85 min was observed from day 4 to day 6 (Figure 3B shows the peak on day 4). MS analysis of the identified peak showed a [M-H]^−^ ion at *m*/*z* 196.05442, which matched the molecular weight of the L-DOPA standard.

### 3.2. Characterization of BP

#### 3.2.1. BP Extraction and Its UV-Vis Light Absorption Spectrum

**Figure 4:**
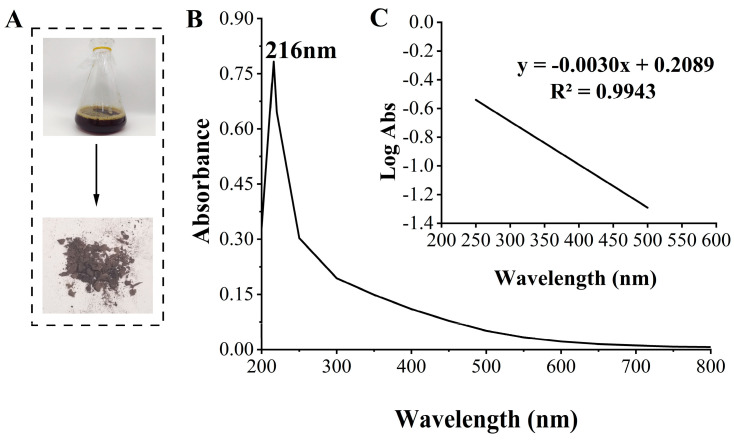
Extraction, purification, and ultraviolet–visible (UV-Vis) absorption spectra of BP. The extracted and purified BP from fermentation broth (**A**), the UV-Vis spectra of purified BP (**B**), and the linear curve obtained after plotting the wavelengths against the logarithmic values of absorbances (**C**).

The purified BP produced by the FBFS 97 strain, according to the extraction and purification procedure outlined in Figure 1, is shown in Figure 4A. Melanin has a strong UV-absorption capacity, which stems from its complex conjugated molecular structure that can effectively absorb and scatter UV photons [2]. To confirm that BP is melanin, the UV-Vis absorption spectrum of the purified BP was measured over a range of 200 nm to 800 nm, revealing a maximum absorption wavelength at 216 nm (Figure 4B). When plotting wavelength against the logarithm of absorbance, a linear curve with a slope of −0.0030 was obtained (Figure 4C), consistent with the typical characteristics of melanin [33]. Furthermore, no significant absorption peaks were observed at 260 nm or 280 nm, indicating that the purified BP did not contain proteins, peptides, and nucleic acid.

#### 3.2.2. Thermal Stability of BP

To further investigate whether the BP produced by the FBFS 97 strain is melanin, its thermal stability was detected through thermogravimetric (TG) and derivative TG (DTG) curves.

**Figure 5:**
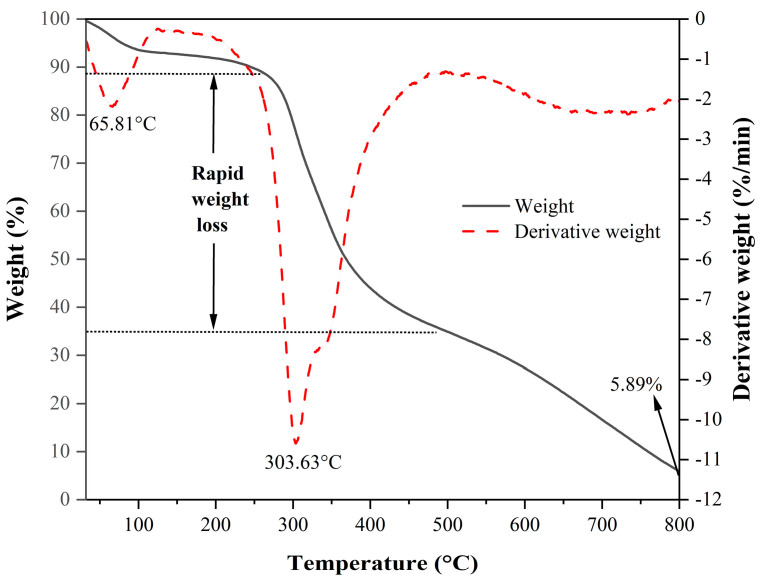
TG curve (black curve) and DTG curve (red curve) of BP.

As shown in Figure 5, the TG curve of BP from the FBFS 97 strain exhibited a gradual decrease in weight as the temperature increased, while its DTG curve displayed multiple peaks, indicating the points of maximum weight change rate. The first peak occurred at 65.81 °C due to the evaporation of weakly and strongly bound water, followed by a pronounced peak at 303.63 °C, which was associated with rapid weight loss because of the release of carbon dioxide [34]. After this stage, the residual weight of BP was 35.24% of its initial quantity. The main thermal degradation occurred at elevated temperatures, approximately 700 °C, mainly attributed to decarboxylation [35]. At the maximum temperature of 800 °C, the weight was further reduced to 5.89% of the initial BP quantity.

#### 3.2.3. Py-GC-MS Analysis of BP

Based on the BP pyrolysis temperature from its TG and DTG curves in Figure 5, the structural information of BP from the FBFS 97 strain was analyzed by Py-GC-MS, which is usually utilized to provide the primary components for high molecular weight materials with a heterogeneous nature such as melanin [36].

As shown in Table 1, the thermal decomposition products of the BP were broken down into benzene (8 and 9), benzonitrile (15), phenol (11), indole (18), furan (4, 7, 23, and 24), pyrrole (20 and 21), pyridine (12), pyrazole (5), oxazole (25), aldehyde (1, 2, and 3), amide (14), indolizine (16), alkane (19, 22, and 26), alkene (10 and 17) and alcohol (13). Among them, benzene, phenol, indole, pyrrole, and their derivatives were the prominent thermal degradation products, aligning with the characteristic profiles of eumelanin [2]. Notably, no heterocyclic or sulfur-containing compounds associated with pheomelanin were detected among the degradation products.

All the above results demonstrate that the BP produced by FBFS 97 is eumelanin, a type of melanin.

### 3.3. Functions of the Genes Relative to Eumelanin Biosynthesis in the FBFS 97 Strain

#### 3.3.1. Prediction and Analysis of the Relative Genes to Eumelanin Biosynthesis

Based on the results mentioned above in Section 3.1 and Section 3.2, it is confirmed that the BP produced by the FBFS 97 strain is eumelanin through the L-DOPA pathway (Figure S1). Therefore, genes related to the L-DOPA pathway for eumelanin biosynthesis were predicted and analyzed based on the FBFS 97 strain’s genome.

It is well known that eumelanin is biosynthesized through the hydroxylation of tyrosine to L-DOPA by the enzyme tyrosinase, followed by oxidation to DOPA quinone. Under oxidative conditions, DOPA quinone can undergo cyclization to form indole quinone. Finally, indole quinone or its carboxylated derivatives spontaneously polymerize to form eumelanin (Figure S1) [15]. During these processes, tyrosinase is a key enzyme (Figure S1) [37], so the conserved domain of tyrosinase (pfam00264) was used to scan the genomes of the FBFS 97 strain and other acetic acid bacteria (AAB) strains in the NCBI database to identify genes related to tyrosinase. Unfortunately, no typical tyrosinase domain was found in the genomes of any AAB strain available in the NCBI database, including that of the FBFS 97 strain. Therefore, it is hypothesized that a laccase (pfam00394, pfam07731, pfam07732, and pfam02578) present in the FBFS 97 strain’s genome may contribute to L-DOPA melanin biosynthesis since the laccase is also a key enzyme involved in the melanin biosynthesis in some bacteria like *Azospirillum lipoferum* [38,39], *Sinorhizobium meliloti* [40], *Bacillus subtilis* [41], and *Bacillus weihenstephanensis* [42]. Finally, a gene of laccase-like protein YfiH containing the conserved domain of laccase (pfam02578) was discovered in the FBFS 97 strain, which may be essential for L-DOPA melanin biosynthesis in this strain.

Considering that phenylalanine can be metabolized into tyrosine under the catalytic action of Phenylalanine hydroxylase (PhhA), promoting eumelanin biosynthesis [43], the genes encoding PhhA and its auxiliary protein, PhhB, were predicted from the FBFS 97 genome. The results showed that PhhA was not found, but its auxiliary protein, PhhB, was identified. Therefore, the *phhB* gene was knocked out to investigate its impact on eumelanin biosynthesis. Moreover, Prephenate dehydratase (PheA) catalyzes the conversion of prephenate to phenylpyruvate, which is further converted to phenylalanine [44]. Based on this, the effect of deleting the Prephenate dehydratase (PheA) gene, *pheA*, on eumelanin biosynthesis in FBFS 97 was also explored.

#### 3.3.2. Studies on the Roles of pheA, yfiH, and phhB in Eumelanin Biosynthesis

**Figure 6:**
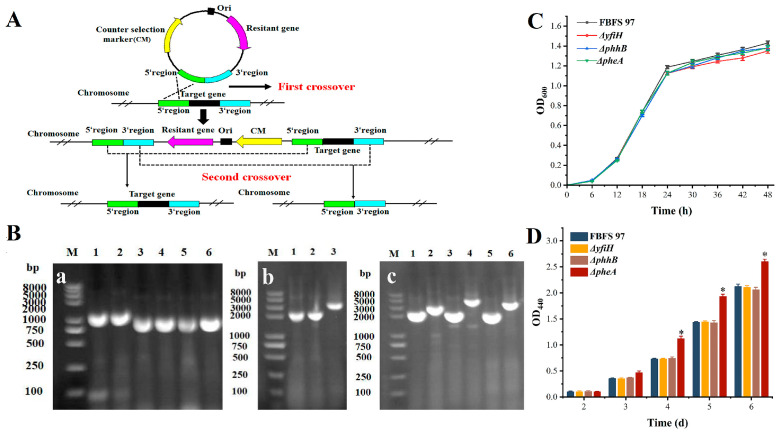
The schematic diagram of gene traceless modification system (**A**), agarose gel electrophoresis of the target gene to be knocked out (**B**): (**a**) agarose gel electrophoresis of the upstream and downstream flanking regions of the target gene to be knocked out (M: DL8000 DNA marker; 1, 2, 3, 4, 5, 6: PCR products of upstream and downstream regions of *yfiH, phhB*, and *pheA*), (**b**) agarose gel electrophoresis of *yfiH*, *phhB*, and *pheA* single-crossover mutants, (**c**) agarose gel electrophoresis of Δ*yfiH*, Δ*phhB*, and Δ*pheA* mutants, cell growth (**C**) and melanin yield (**D**) of wild-type FBFS 97 and the mutant strains. *: *p* < 0.05.

According to the predicted results in Section 3.3.1, three genes potentially related to eumelanin production, including *yfiH* encoding YfiH, *phhB* encoding PhhB, and *pheA* encoding PheA from the genome of *G. tumulisoli* FBFS 97, were deleted using gene traceless modification system, respectively (Figure 6A). The gel electrophoresis results of the mutant strains (Δ*yfiH*, Δ*phhB*, and Δ*pheA*) are shown in Figure 6B. Especially, the upstream and downstream homology arms of the *yfiH, phhB*, and *pheA* genes were amplified using primers using FBFS 97 genomic DNA as a template, and the validation plots of the specific bands obtained from the amplification are shown in Figure 6B(a). The sizes of the products were as expected (Table S1). Subsequently, the colonies grown on kanamycin (50 μg/mL) plates after electro-transformation were picked, and the surviving colonies were selected for colony PCR verification after three passages on the same kanamycin-resistant plates. The results of colony PCR are shown in Figure 6B(b). The band sizes were correct, lanes 1 and 2 corresponded to the upstream integration of *yfiH* and *phhB* single-crossover transformants, and lane 3 corresponded to the downstream integration of *pheA* single-crossover transformants, which can be used for the subsequent reverse screening experiments. Finally, the colonies grown on the reverse screening markers 5-fluorocytosine (5-FC) plate were delineated on the kanamycin plate, and the colonies that could not grow on the kanamycin plate were picked for colony PCR verification. The results of the knockout strains are shown in Figure 6B(c). The sizes of bands 1, 3, and 5 were 2256, 2213, and 2177 bp, corresponding to the PCR products of Δ*yfiH*, Δ*phhB*, and Δ*pheA*, respectively. The sizes of bands 2, 4, and 6 were 3071, 2597, and 3213 bp, corresponding to the PCR products of the wild-type FBFS 97, with the size differences matching the size of the genes to be knocked out, indicating that the *yfiH*, *phhB*, and *pheA* knockout strains were successfully obtained.

Each genotype strain was inoculated into the GYE seed medium, and their growth was monitored. As presented in Figure 6C, the growths of Δ*yfiH*, Δ*phhB*, and Δ*pheA* were essentially the same as that of the FBFS 97 strain, suggesting that the knockout of the three genes did not impact the growth of the FBFS 97 strain.

To further investigate the roles of *yfiH*, *phhB*, and *pheA* in melanin production, melanin production assays were conducted for each mutant. The strains were cultured in GYE fermentation media, and the OD_440_ values of the supernatant from their fermentation broth were measured. As illustrated in Figure 6D, there was no significant difference in OD_440_ values between the mutants and the wild-type strain during the initial three days. However, the OD_440_ values of the Δ*pheA* mutant showed significant differences from the fourth day to the sixth day (*p <* 0.05) compared to the wild type and other mutants. These results suggest that *pheA* knockout enhances melanin production.

About *yfiH*, after it was deleted, the OD_440_ values of Δ*yfiH* were the same as those of the wild-type FBFS 97 strain (Figure 6D), suggesting that YfiH is not a relative enzyme with the eumelanin biosynthesis in the FBFS 97 strain. Chai et al. (2017) [19] also observed *yfiH* deletion mutant did not affect the melanin production in *Aeromonas media* WS. With regard to *phhB*, when it was deleted, the OD_440_ values of Δ*phhB* did not change compared to those of the wild-type FBFS 97 strain (Figure 6D), indicating that PhhB is not the relative enzyme to melanin production.

## 4. Discussion

**Figure 7:**
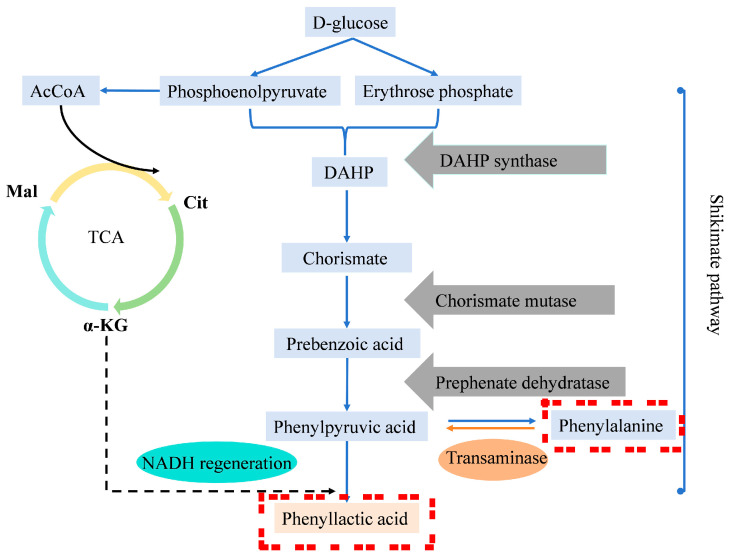
Pathway for biosynthesis of phenyllactic acid.

Most AAB strains can synthesize a large amount of pigments, which are generally applied as their taxonomic indicators [21]. *G. tumulisoli* FBFS 97, which can produce a large amount of BP, was isolated in our previous research [22]. Notably, the BP production of the FBFS 97 strain was significantly increased in the presence of tyrosine (Figure 2D), suggesting that BP may be associated with melanin, as it has been demonstrated that the addition of tyrosine to the media increased melanin production in many bacteria, such as *Streptomyces antibioticus* [45], *Bacillus thuringiensis* [46], and *Aeromonas media* WS [47]. However, the addition of phenylalanine did not increase BP production (Figure 2D), possibly due to the phenylalanine being diverted to other metabolic pathways, such as the phenyllactic acid biosynthesis pathway (Figure 7) [48]. This result also suggests that FBFS 97 may not rely on phenylalanine metabolism to provide the precursor tyrosine for melanin biosynthesis.

At the same time, we found that when D-glucose was used in the carbon source experiments, BP levels were the highest, while glycerol resulted in the lowest BP production (Figure 2A). We also found that BP production was highest when 4% D-glucose was used as the carbon source (Figure 2B). The higher BP production observed with D-glucose as a carbon source may be attributed to its efficient metabolism, which supports BP biosynthesis. Additionally, glucose is metabolized to produce intermediates that are key to BP synthesis [49]. A study found that pigment produced by *Aeromonas salmonicida* 34mel^T^ was inhibited when 1% glycerol was used as a carbon source [50]. However, since other carbon sources also influenced BP formation to some extent, further research is required to explore the underlying mechanisms.

L-DOPA, a key melanin intermediate [47,51,52], was successfully detected in the BP fermentation broth of the FBFS 97 strain (Figure 3B), indicating that BP is synthesized through the DOPA-based pathway and may be melanin. The UV-Vis spectrum results have shown that BP exhibits a maximum peak in the ultraviolet range, and its absorbance decreases with increasing wavelength (Figure 4B). When plotting wavelength against the logarithm of absorbance, a linear curve with a slope of −0.0030 was obtained (Figure 4C). This behavior is in strong agreement with the properties of melanin [53,54], further supporting the conclusion that BP is melanin. Moreover, the purified BP showed good thermal stability (Figure 5). Similar thermal degradation results were observed in previous research on melanin [34,55], which suggests that BP may be melanin. Melanin’s thermal stability, a distinguishing feature compared to other natural polymers, is attributed to its graphite-like polymeric structure [56]. Interestingly, compared with eumelanin, pheomelanin, a sulfur-containing melanin, showed an extremely pronounced exothermic peak in the final stage of thermal degradation due to the decomposition of sulfur units present in its structure [57]. In the current study, no such additional peak was observed, suggesting that the BP produced by the FBFS 97 strain does not contain sulfur-containing units, so the BP is more likely to be eumelanin. Due to its heterogeneous nature, melanin lacks a well-defined unique structure [56]. Py-GC-MS is a powerful technique that provides valuable structural insights for natural products, especially for those with high molecular weights and heteropolymeric structures, like melanin [36]. The Py-GC-MS results of the purified BP showed the presence of eumelanin cleavage products, without detectable pheomelanin cleavage products (Table 1), suggesting that BP is eumelanin.

However, further analysis revealed that the genetic basis for melanin biosynthesis in the FBFS 97 strain differs from known melanin pathways. Typically, the most common catalytic enzyme involved in the biosynthesis of eumelanin is tyrosinase, which plays a key role in bacterial eumelanin biosynthesis, involving the oxidation of tyrosine and L-DOPA [43,58]. FBFS 97 is believed to synthesize eumelanin via the L-DOPA pathway. Notably, no gene of typical bacterial tyrosinase was found in the genome of the FBFS 97 strain, consistent with the findings of Chai et al. (2012) [59]. Therefore, the fact that no tyrosinase gene was detected in the genome of the FBFS 97 strain raises an important question about the eumelanin biosynthetic mechanism.

In addition, laccase can also catalyze the biosynthesis of eumelanin, which can oxidize L-DOPA to produce eumelanin [19,60,61]. Further studies in this research revealed that knocking out the *yfiH* gene, which encodes a laccase-like protein potentially involved in melanin biosynthesis in the FBFS 97 strain, did not alter melanin content. This suggests that eumelanin biosynthesis in FBFS 97 is not dependent on this laccase-like protein. Typically, phenylalanine is metabolized to tyrosine by the enzyme PhhA and its auxiliary protein PhhB [62]. Since we did not find *phhA* in the FBFS 97 genome but found *phhB*, we knocked out *phhB* to investigate its potential effect on eumelanin biosynthesis. The results showed that knocking out *phhB* did not affect eumelanin production in the FBFS 97 strain (Figure 6D), suggesting that *phhB* may need to synergize with *phhA* to exert its regulatory or co-functional role, which in turn affects eumelanin synthesis. Therefore, we speculate that the phenylalanine-to-tyrosine metabolic pathway is unimportant in FBFS 97, and tyrosine production may not rely on phenylalanine. Notably, we observed that the *pheA* knockout strain showed a marked increase in eumelanin production after four days of cultivation (Figure 6D). A similar result was also observed in *Escherichia coli* BL21, where the knockout of *pheA* led to a 1.29-fold increase in tyrosine production [63]. Different enzymes can convert prephenate into either tyrosine or phenylpyruvate [64]. PheA catalyzes the conversion of prephenate to phenylpyruvate, which is further converted to phenylalanine [65]. The knockout of *pheA* may result in more prephenate being converted to tyrosine, thereby increasing the synthesis of endogenous tyrosine, an important precursor for eumelanin biosynthesis [66], which subsequently promotes eumelanin production. Additionally, *pheA* knockout may also affect other pathways through metabolic network regulation, providing additional support for eumelanin biosynthesis, and the underlying mechanism warrants further exploration.

**Figure 8:**
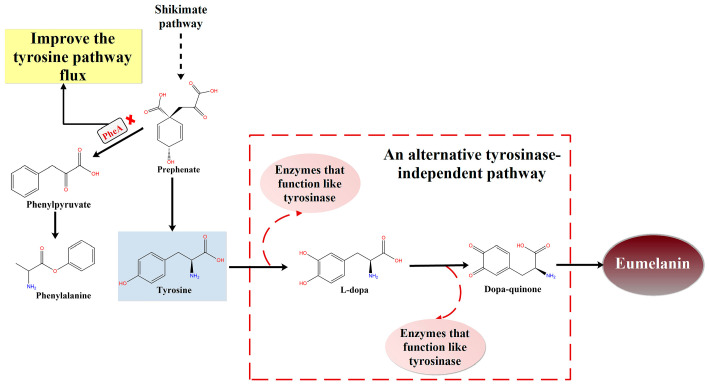
Proposed eumelanin biosynthesis pathway in the FBFS 97 strain. (The red cross indicates the knockout of *pheA*, which increases tyrosine flux and promotes eumelanin production. The red dashed box indicates that eumelanin formation in FBFS 97 may involve enzymes that function like tyrosinase, requiring further study).

In summary, the results of this study suggest that the FBFS 97 strain may convert tyrosine to eumelanin through an alternative tyrosinase-independent pathway (Figure 8). To further investigate this hypothesis, based on the literature [67], we used a web platform, namely, ECRECer (https://ecrecer.biodesign.ac.cn, accessed on 17 October 2024) to search for enzymes with similar functions for eumelanin biosynthesis, and the results are listed in Table S2. Notably, this tyrosinase-independent pathway may involve other oxidative enzymes instead of classical tyrosinase, such as peroxidase or catalase, which are involved in melanin biosynthesis in bacteria [19,68,69]. In the future, it is necessary to identify the specific enzymes responsible for eumelanin biosynthesis in the FBFS 97 strain through biochemical assays and enzyme activity measurements.

## 5. Conclusions

In the current study, the BP produced by *Gluconacetobacter tumulisoli* FBFS 97 was identified as eumelanin, which was biosynthesized via the L-DOPA melanin biosynthesis pathway using tyrosine as a precursor. Eumelanin-related genes (*yfiH*, *phhB*, and *pheA*) were found in the FBFS strain’s genome, and their knockout mutants were constructed. Knocking out the *pheA* gene led to an increase in eumelanin production, providing new targets for regulating eumelanin biosynthesis. However, no tyrosinase-related gene was discovered from the FBFS 97 genome. Although the *yfiH* gene encoding a laccase-like protein relevant to eumelanin synthesis was identified in the FBFS 97 genome, its knockout did not affect eumelanin production. Based on these findings, a potential pathway for eumelanin biosynthesis in the FBFS 97 strain is proposed.

## Figures and Tables

**Table 1:** Py-GC-MS analytical products of BP.

No.	Compound Structure	Retention Index	No.	Compound Structure	Retention Index
1	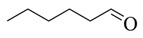	Hexanal 806	14	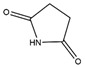	Succinimide 934
2	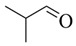	Isobutyraldehyde 543	15	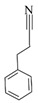	Phenylpro-pionitrile 1238
3	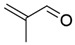	Dimethacryrolein 574	16	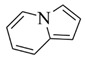	Indolizine 991
4	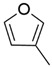	Trimethylfuran 642	17	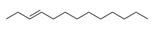	3-Tridecene 1321
5	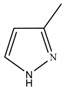	Trimethylpyrazole 921	18	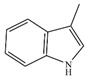	3-Methylindole 1264
6	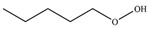	Amyl hydrogen peroxide 878	19	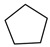	Cyclopentane 600
7	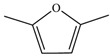	2,5-Dimethylfuran 732	20	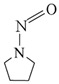	N-Nitrosopyrrolidine 1047
8	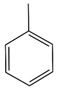	Methylbenzene 794	21	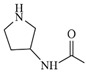	Acetamidopyrrolidine 1306
9	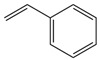	Styrene 883	22	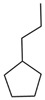	n-Propylcyclopentane 859
10	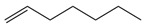	1-Heptene 707	23	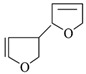	2-(2,3-dihydrofuran-3-yl)-2,5-dihydrofuran 1044
11	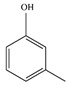	m-Cresol 1014	24	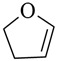	2,3-Dihydrofuran 571
12	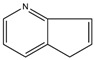	5H-Cyclopenta[b]pyridine 1023	25	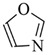	Oxazole 591
13	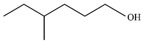	4-Methyl-1-hexano l896	26	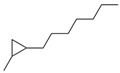	1-Heptyl-2-methylcyclopropane 1078

## Data Availability

The original contributions presented in this study are included in the article/Supplementary Material. Further inquiries can be directed to the corresponding author.

## Supplementary Materials

The following supporting information can be downloaded at: https://www.mdpi.com/article/10.3390/microorganisms13030480/s1, Figure S1: Melanin biosynthetic pathways commonly found in bacteria include four different classes: eumelanin, pheomelanin, pyomelanin, and 4-hydroxyphenylacetic acid melanin. Table S1: Primers used in this study. Table S2: prediction of enzymes that may function like tyrosinase in the FBFS 97 genome.
