# Supplementary material for: Investigation of Eumelanin Biosynthesis in Gluconacetobacter tumulisoli FBFS 97: A Novel Insight into a Bacterial Melanin Producer
Source: Microorganisms. 2025 Feb 21;13(3):480. doi: 10.3390/microorganisms13030480 (PMC11944832; doi:10.3390/microorganisms13030480)
Supplement: Supplementary file 1 [file microorganisms-13-00480-s001.zip › microorganisms-3460502-supplementary.pdf]

Supplementary materials for
Investigation of Eumelanin Biosynthesis in *Gluconacetobacter tumulisoli* FBFS 97: A Novel Insight into a Bacterial Melanin Producer

Jiayun Song [1,2,†], Yanqin Ma [1,2,†], Zhenzhen Xie [1,2] and Fusheng Chen [1,2,3,4,*]

1 College of Food Science and Technology, Huazhong Agricultural University, Wuhan430070, China; jiayunsong@webmail.hzau.edu.cn (J.S.); mayq@webmail.hzau.edu.cn (Y.M.); 13683977281@163.com (Z.X.)
2 National Key Laboratory of Agricultural Microbiology, Huazhong Agricultural University, Wuhan 430070, China
3 Hubei International Scientific and Technological Cooperation Base of Traditional Fermented Foods, Huazhong Agricultural University, Wuhan430070, China
4 School of Live Science, Guizhou Normal University, Guiyang 550025, China
* Correspondence: chenfs@mail.hzau.edu.cn (F.C.); Tel.: +86-13986273471
† These authors have contributed equally to this work

This document includes the following content:

1. **Figure S1**
2. **Table S1 and Table S2**

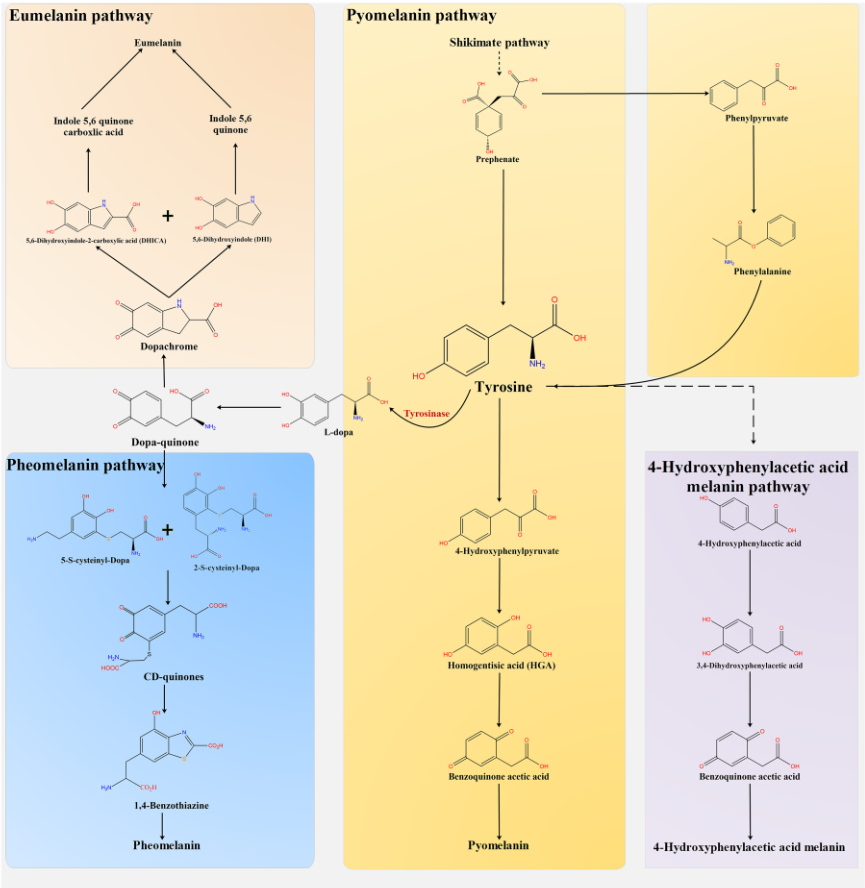

Figure S1. Melanin biosynthetic pathways commonly found in bacteria include four different classes: eumelanin, pheomelanin, pyomelanin, and 4-hydroxyphenylacetic acid melanin.

Table S1. Primers used in this study.

Primer ID	Sequence	Function
yfiH-upF1	CAGCTATGACATGATTACGAATTCG-CAACTCGGACGCAACCTTC	Amplification of the 5' UTR of *yfiH* for traceless gene modification (1033 bp).
yfiH-dwR1	GCCATGCCATGACCGATAACGGGAT-TCCGGACGAGTCGAT	
yfiH-upF2	ATCGACTCGTCCG-GAATCCCGTTATCGGTCATGGCATGGC	Amplification of the 3' UTR of *yfiH* for traceless gene modification (1023 bp).
yfiH-dwR2	GCATGCCTGCAGGTCGACTCTAGA-CAGGTGCTGTGCGTTCCCAT	
check- *yfiH*F	CTTCTCGGCCAGAGGCAGGT	Amplification of the fragment with this primer pair to confirm the *yfiH* knockout strain construction. The Δ*yfiH* is 2256 bp, while the wild-type is 3071 bp.
check- *yfiH*R	TGTGCGTTCCCATGGCGATC	
pheA-upF1	CGGAATTCGTGG-CAATCATGCCGCTCTT	Amplification of the 5' UTR of *pheA* for traceless gene modification (945 bp).
pheA-dwR1	CAGGTGCACGATGAACGTCGACGCAC-GACATCAAATGGAAAC	
pheA-upF2	GTTTCCATTTGATGTCGTGCGTCGAC-GTTCATCGTGCACCTG	Amplification of the 3' UTR of *pheA* for traceless gene modification (934 bp).
pheA-dwR2	GCTCTAGACATCCGGAATACGTT-GCTCCAC	
check- *pheA*F	ACTTGTCCACCTGCTTGCCAT	Amplification of the fragment with this primer pair to confirm the *pheA* knockout strain construction. The Δ*pheA* is 2177 bp, while the wild-type is 3213 bp.
check- *pheA*R	GGCGGCTCCAATACGGTTG	
phhB-upF1	CAGCTATGACATGATTAC-GAATTCTCATGATCCGGACATGGAGC	Amplification of the 5' UTR of *phhB* for traceless gene modification (1004 bp).
phhB-dwR1	CGTAGCAAGACCGGGG-CAAACTTCGCCCAAGCCCACCAGG	
phhB-upF2	CCTGGTGGGCTTGGGCGAAGTTT-GCCCCGGTCTTGCTACG	Amplification of the 3' UTR of *phhB* for traceless gene modification (1019 bp).
phhB-dwR2	GCATGCCTGCAGGTCGACTCTA-GATGAAGATCGAGGCCAGCGAG	
check- *phhB*F	TCATTTCGGTCGGTTTCACG	Amplification of the fragment with this primer pair to confirm the *pheA* knockout strain construction. The Δ*phhB* is 2213 bp, while the wild-type is 2597 bp.
check- *phhB*R	ATCGCGCGCGAGGAAGACT	

Table S2. Prediction of enzymes that may function like tyrosinase in the FBFS 97 genome.

Gene ID	Annotation	EC number (predicted by ECRECer)	Annotation (predicted by ECRECer)	E value*
0336	hypothetical protein	1.11.1.15	peroxidase	0.99149
0981	hypothetical protein	1.11.1.-	peroxidase	1.0
1399	hypothetical protein	1.11.1.-	peroxidase	1.0
1823	hypothetical protein	1.11.1.15	peroxidase	0.93547
1839	hypothetical protein	1.11.1.15	peroxidase	0.99999
2279	hypothetical protein	1.11.1.15	peroxidase	1.0
2302	hypothetical protein	1.11.1.15	peroxidase	1.0
2606	hypothetical protein	1.11.1.15	peroxidase	1.0
2607	hypothetical protein	1.11.1.15	peroxidase	0.99999
2616	hypothetical protein	1.11.1.15	peroxidase	1.0
2667	hypothetical protein	1.11.1.6	catalase	1.0
3000	hypothetical protein	1.11.1.15	peroxidase	1.0

*: The value closer to 1 indicates a more reliable prediction.
